# Adenoid basal lesions of the uterine cervix: evolving terminology and clinicopathological concepts

**DOI:** 10.1186/1746-1596-1-18

**Published:** 2006-08-15

**Authors:** Michael J Russell, Oluwole Fadare

**Affiliations:** 1 Department of Pathology, Wilford Hall Medical Center, Lackland AFB, TX, USA; 2 San Antonio Uniformed Services Health Education Consortium Residency Program in Pathology, San Antonio, TX, USA; 3 Department of Pathology, University of Texas Health Sciences Center at San Antonio, San Antonio, TX, USA

## Abstract

The epithelial proliferations that are designated *adenoid basal carcinoma *(ABC) in the current classification from the World Health Organization represent <1% of all cervical malignancies. These lesions may be associated, and occasionally show morphologic transitions with, conventional cervical malignancies. The determination of the precise frequency with which these so-called ABCs show this association is hampered by the inherent selection bias in the reported cases. However, this frequency appears to be substantial (>15%). The biologic course of ABCs that are associated with separate malignancies is largely dependent on the clinicopathologic parameters of the associated malignancies. Morphologically pure lesions, in contrast, have largely been associated with favorable patient outcomes, as none of the 66 reported patients have experienced tumor recurrence, metastases or tumor-associated death, irrespective of the modality of treatment. Although the finding of genome integrated high-risk human papillomavirus (HPV) types and p53 alterations in adenoid basal lesions (ABL) argue in support of their neoplastic nature, we identified no lines evidence that suggest an inherent malignancy for morphologically pure lesions. The finding of morphologic transitions between ABLs and conventional malignancies and shared HPV types in these areas, suggest that ABLs have some malignant potential. However, the precise magnitude of this potential is not readily quantifiable and should not dictate the management of morphologically pure lesions that are entirely evaluable. ABLs continue to occupy a unique position in human oncology in which the term carcinoma (without an in-situ suffix) is applied to a tumor that has not been shown to recur, metastasize or cause death. We concur with a previous proposal that the term ABC should be discarded and replaced with *Adenoid Basal Epithelioma *(ABE). In our opinion, there is insufficient evidence at present time to expose patients with morphologically pure lesions to the ominous implications – social, psychological, medical, financial – of a "carcinoma" diagnosis. Morphologically impure lesions should not be designated ABC or ABE. Furthermore, given the uncertainties regarding the frequency with which ABE are associated with separate malignancies, we suggest that the ABE designation only be applied when the tumor in question is entirely evaluable e.g in a hysterectomy specimen or in an excisional biopsy with negative margins. Otherwise, the generic designation *Adenoid Basal Tumor *is preferable. This approach strikes an appropriate balance between the need to prevent over-treatment of pure lesions on one hand, and the need to ensure that the lesions are indeed pure on the other.

## Background

The historical evolution of the lesions that are currently designated "adenoid basal carcinomas" [1-25] is inextricably linked to adenoid cystic carcinoma [26-84], a diagnostic entity under which it was subsumed for many years prior to and even after its delineation, and a tumor with which it shares some morphologic features and probably, a histogenetic basis. Adenoid cystic carcinoma of the cervix (ACC) was originally described as "cylindroma" by Paalman and Counsellor [84] in 1949. Fifteen years later, Moss and Collins [83] described a distinctive cervical neoplasm that was predominantly comprised of ACC but which also contained foci of small basaloid nests. The authors designated this tumor "adenoid cystic basal carcinoma". However, the 1966 report of Baggish and Woodruff [24], in which the term "adenoid-basal carcinoma" (ABC) was initially used, is widely credited [85-89] with the delineation of this lesion as a separate clinicopathologic entity. The latter authors described 3 examples of a morphologically distinctive cervical tumor that was comprised of small basaloid cells nests that seemed to be "dropping off" the basal layer of the overlying surface epithelium, which did not elicit any significant stromal reaction, and which in all 3 cases, were associated with carcinoma-in-situ of the overlying epithelium. All 3 patients were treated with surgical resections and none had experienced recurrences within the follow-up period. Based in part on the absence of a significant stromal reaction, the authors suggested that the lesions were "only as locally invasive as the basal cell lesion of the skin and demand no more radical therapy than wide local excision" [24]. In a follow-up report [23], all 3 patients were alive and well 5–13 years after their hysterectomies. In the same report, Baggish and Woodruff reported 5 additional cases, introducing the term "adenoid basal hyperplasia" [23] as well as the concept that different malignancies may be associated with ABC. In 1972, Shilkin [73] reported a cervical tumor which he designated ABC. The author's photomicrographs showed, in our opinion, an archetypal example of ACC. However, a histochemical analysis of the lesion showed it to be devoid of myoepithelial cells, in contrast to ACCs elsewhere. Thus, the author did not consider the lesion an example of ACC and designated the lesion an ABC. The ABC/ACC distinction continued to be very nebulous in the middle to late 1970s. The 1975 report of Russell [22] suggested that the author considered the terms to be interchangeable. In the 1980 series of ACC reported by Prempree et al [58], clear examples of ABC are included, and at least one case of ABC (case 3) is included in the series of cervical "basaloid carcinoma" reported by Daroca and Dhurandhar [25] that same year. It was not until the publication of two important reports in 1985 [21] and 1988 [20] that the important clinicopathologic differences between ACC and ABC were clarified. Subsequent reports have thus been able to focus attention on the many important aspects of true ABC that remain to be understood, including histogenesis, precise malignant potential, and other areas that are still lacking in consensus, including nomenclature. In this review, the descriptive term *adenoid basal lesion(s) *(ABL) is used to generically refer to the full spectrum of adenoid basal lesions that that have been variably described in the literature as adenoid basal hyperplasia, ABC (pure lesions and those associated with separate malignancies) and adenoid basal epithelioma, unless otherwise specified. A comprehensive clinicopathologic review of all reported cases of ABL in the english-language literature is presented, and we discuss our practical approach to these enigmatic lesions.

### Incidence

There are no studies that have systematically investigated the incidence of ABL. However, retrospective analyses by most authors suggest that pure ABL constitute less than 1% of all cervical malignancies [85]. Teramoto et al [2] identified 1 pure ABL out of 2600 "resected cervical malignancies" diagnosed at the Shikoku Cancer Center (Japan). In contrast, a study emanating out of Women's Center in Australia identified 5 cases out of 106 (4.7%) cervical cancers diagnosed over a 25–26-month period [17]. A search of the computerized database of a large academic medical center in Northeast United States yielded only 4 cases coded as *adenoid basal *out of 189 cervical carcinomas diagnosed over a 7-year period (O Fadare, unpublished data, February 2005).

#### Clinical features of patients with pure adenoid basal lesions

The 66 previously reported morphologically pure ABL in the English literature are summarized in table 1. Cases associated with "separate malignancies" (vide infra) have been excluded. However, we elected to keep 3 cases with microinvasive carcinoma, because the latter only develops in the setting of high grade dysplasia – a nearly ubiquitous finding in these patients – and because their presence is not expected to alter the natural history of the ABL any more than the high grade dysplasia alone. For the 52 patients in which this information was itemized, patient age ranged from 21 to 91 years (mean 64.6; Median 66.5). Although some of the earlier series [23,24] as well as those emanating out of South Africa [7,9] seemed to suggest a marked predilection for black females, others have found no such predilection [20,21]. However, ABL does seem to disproportionately affect non-white women. Approximately 80% of patients were asymptomatic at initial diagnosis, and 80–90% of them came to clinical attention due to an abnormal pap smear; the latter was a high-grade squamous intraepithelial lesion in >90% of cases. A pelvic examination fails to reveal an abnormality in almost all cases of pure ABL. Similarly, pathologic evaluation of the hysterectomy or conization specimens usually fails to reveal a mass lesion. Cervical ulceration, hemorrhage or eversion was identified in 7 cases, and a mass lesion was noted in 2 [13].

**Table 1:** Previously reported cases of morphologically pure adenoid basal lesions in the English literature.

	**Authors (reference)**	**No of patients**	**age (yrs)**	**Race**	**Presentation/pap smear findings**	**Gross or pelvic examination findings**	**Associated cervical intraepithelial dysplastic lesion**	**Diagnostic and/or Therapeutic measure(s)**	**Lymph node status**	**Follow-up, (months)**
1	Baggish & Woodruff [24]***	1	63	white	Vaginal bleeding/NR	Atrophic vaginal mucosa; NCF	CIS	Hysterectomy/BSO	NPR	NED 156
2		1	62	black	Uterine prolapse/NR	NCF	CIS	Hysterectomy	NPR	NED 84
3		1	39	black	Abnormal pap smear	NR	CIS	Conization/Hysterectomy	NPR	NED 60
4	Baggish & Woodruff [23]	1	60	white	"incidental"/negative	NCF	None	Hysterectomy/BSO	NPR	NED 6
5	Cases 1–3	1	80	white	"incidental"/negative	NCF	None	Hysterectomy/BSO	NPR	NED 6
6		1	80	black	Lower abdominal pressure/negative	Cervical eversion	CIS	Conization/Hysterectomy/BSO	NPR	NED 6
7	Russell & Laverty [22]	1	62	NR	uterine prolapse and vaginal bleeding/"Very Atypical cells"	Cervical ulceration	None	Hysterectomy	NPR	NR
8	Daroca & Dhurandhar [25], Case 3	1	60	NR	Post menopausal bleeding	Cervical erosion	CIS	Hysterectomy/BSO	NPR	NED 12
9–13	van Dinh & Woodrhuff [21]##	5	mean 56	white 3, black 2	Vaginal bleeding n = 1, asymptomatic n = 4/moderate atypia n = 5	Cervical ulceration n = 1, NCF n = 4	CIN3 n = 3, None n = 2	dilatation and curettage n = 1, Hysterectomy +/- BSO n = 4	NPR	DOC n = 1, NED 24–144, n = 4
14	Ferry & Scully [20]	1	69	black	Abnormal pap smear n = 11, PMB n = 2	NCF n = 10, cervical induration n = 2,	CIS	Hysterectomy/BSO	NPR	NED 60
15	Cases 1–13	1	63	NR			CIS	Hysterectomy/BSO	NPR	NED 120
16		1	66	black			CIS	Cervical excision/Rad	NPR	DOC 24
17		1	75	white			CIS	Hysterectomy/BSO	NPR	NED 84
18		1	65	asian			CIS	Hysterectomy/BSO/Rad	NPR	NED 72
19		1	51	NR			CIS	Hysterectomy/BSO	NPR	NED 24
20		1	56	NR			CIS	Cervical excision	NPR	NED 72
21		1	78	white			CIS	Cervical excision	NPR	NED 72
22		1	59	white			CIS	Hysterectomy/RSO	NPR	NED 60
23		1	67	black			CIS	Hysterectomy/BSO	NPR	NED 48
24		1	57	white			CIS	Hysterectomy/BSO	NPR	NED 24
25		1	72	black			CIS	Conization	NPR	NED 29
26		1	57	NR			CIS	NR	NPR	DOC 0
27	Langlois et al [19]	1	59	NR	Asymptomatic/abnormal pap smear	NCF	CIN3	Conization, Hysterectomy	NPR	NR
28	Peterson & Neumann [18]	1	64	other	Asymptomatic/HGSIL	NR	CIN2	Conization	NPR	NR
29	Layton-Henry et al [17]	1	62	NR	Abnormal Pap smear n = 5/HGSIL, one with "possible invasion", n = 3; inconclusive n = 2	NCF	CIN	Conization, Hysterectomy, BSO, LN	Negative	NED 1
30		1	64	NR		NCF	CIN	Conization, Hysterectomy, BSO	NPR	NED 7
31		1	75	NR		NCF	CIN	Conization	NPR	NED 15
32		1	60	NR		NCF	CIN	Conization, Hysterectomy, BSO, LN	Negative	NED 26
33		1	59	NR		NCF	CIN	Conization, Hysterectomy, BSO, LN	Negative	VAIN 16
34	Powers & Frable [16]	1	79	white	Asymptomatic/SCC	AWL	CIS	Conization, Hysterectomy BSO, LN	Negative	NED 18
35		1	67	black	Presentation @/"severely atypical small cells"	NCF	None	Conization, Rad	NPR	NED 16
36–40	Jones et al [13]	1	Mean 51	white n = 5	abnormal pap n = 4, PMB n = 1	"Ulcerated, irregular indurated mass" n = 2, NCF n = 3	CIN 1 to3 (n = 5)	Hysterectomy/BSO (n = 5), Hysterectomy/BSO/LN (n = 4). One patient also received Rad	Negative	NED 26 Mean
41	Yoshida et al [14]	1	49	asian	Asymptomatic	NCF	CIN 3	Hysterectomy	NPR	NR
42–45*	Grayson et al [9] (Cases 4,5,7,8)	4	NI	black	NI	NI	NI	NI	NI	NI
46	Brainard & Hart [10]	1	91	NR	Dysuria, Night sweats/HGSIL	Focal hemorrhage	CIS	Conization	NPR	NED 4
47		1	68	NR	Asymptomatic/HGSIL	NCF	CIS	Conization, Hysterectomy, BSO, LN	Negative	NED 5
48		1	78	NR	Vaginal spotting/HGSIL	NCF	Severe dysplasia	Conization, Hysterectomy, BSO, LN	Negative	NED 15
49		1	30	NR	Asymptomatic/HGSIL	NCF	Moderate severe dysplasia	Conization, Hysterectomy, BSO, LN	Negative	NED 17
50		1	71	NR	Asymptomatic/HGSIL	NCF	CIS	Conization	NPR	NED 21
51		1	67	NR	Asymptomatic/HGSIL	NCF	Microinvasive Carcinoma##	Conization, Hysterectomy, BSO, LN	Negative	NED 28
52		1	70	NR	Dysuria, Hematuria/Negative	NCF	None	Hysterectomy	NPR	NED 30
53		1	72	NR	Asymptomatic/HGSIL	NCF	CIS	Conization, Hysterectomy	NPR	NED 70
54		1	71	NR	Asymptomatic/HGSIL	NCF	CIS	Conization, BSO, Rad, LN	Negative	NED 82
55		1	76	NR	NR/HGSIL	NCF	CIS	Hysterectomy, BSO	NPR	NED 24
56		1	70	NR	Asymptomatic/LGSIL	NCF	CIS	Hysterectomy, Rad	NPR	NED 63
57		1	83	NR	Asymptomatic/HGSIL	NCF	CIS	Conization	NPR	NED 87
58		1	35	NR	Pelvic mass@/Normal	NCF	None	Hysterectomy, BSO, LN, Chemotherapy [all@]	Negative	NED 49
59		1	57	NR	Asymptomatic/CIS	NCF	CIN	Conization	NPR	NED 5
60		1	69	NR	Asymptomatic/abnormal pap smear	NCF	None	Conization	NPR	NR
61	Senzaki et al [11]	1	69	asian	Vaginal bleeding	NCF	CIS	Hysterectomy, BSO, LN [all@]	Negative	NED 6
62	Hiroi et al [6]	1	74	asian	Vaginal bleeding	NCF	CIN 3	Hysterectomy	NPR	NR
63	Khoury et al [4]	1	79	black	Asymptomatic/HGSIL	NCF	Severe dysplasia	Hysterectomy, BSO	NPR	NR
64	Teramoto [2]	1	69	asian	Asymptomatic/HGSIL	NR	CIN3	Hysterectomy and chemotherapy@	NPR	108
65	Zamecnik & Skrivanek [1]	1	21	NR	Asymptomatic//LGSIL	NCF	CIN1-3	Conization	NPR	NED 1
66	Parwani et al [1]	1	65	NR	NR/HGSIL	NR	Microinvasive Carcinoma##	Conization, Hysterectomy, LN	Negative	NED 78

*Abbreviations: *NED, no evidence of disease; CIS, carcinoma in situ; LN, lymph node dissection; N/A, not applicable; PMB postmenopausal bleeding; BSO or RSO bilateral or right salpingo-oophorectomy; Rad, radiation therapy; NPR Lymph node dissection not performed or reported; NCF no gross cervical findings; DOC death from other causes; LGSIL low grade squamous intraepithelial lesion; HGSIL high grade squamous intraepithelial lesion; CIN cervical intraepithelial neoplasia. Cases in Cviko et al [8] are excluded due to lack of sufficient clincopathological information; SCC squamous cell carcinoma. **Presumes cases in Grayson et al (1997) are the same in Grayson et al (1999); AWL: acetowhite lesions. NI: information not included due to a lack of sufficient itemization in original manuscript. NA: information not available. *excludes the 4 cases in Grayson et al [9] with "divergent neoplastic epithelial differentiation"; *** follow-up as reported in [23]; @ Unrelated to cervical pathology; ## Includes the cases of microinvasive carcinoma [1,10,20], see text

#### Pathologic features of pure adenoid basal lesions

**Figure 1:**
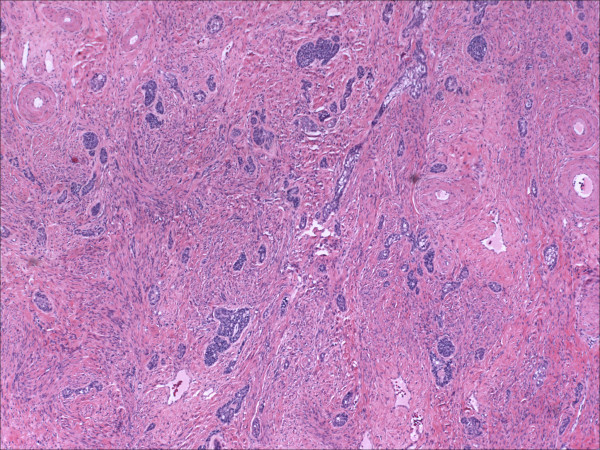
Scanning magnification view of a pure adenoid basal lesion (adenoid basal epithelioma), showing an infiltrative proliferation of basaloid nests. (hematoxylin and eosin, original magnification 10×)

**Figure 2:**
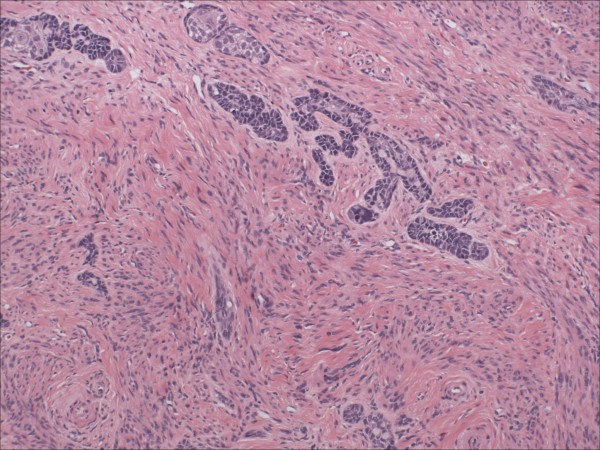
Intermediate power view of adenoid basal nests. Note the lack of any significant stromal reaction. (hematoxylin and eosin, original magnification 20×)

**Figure 3:**
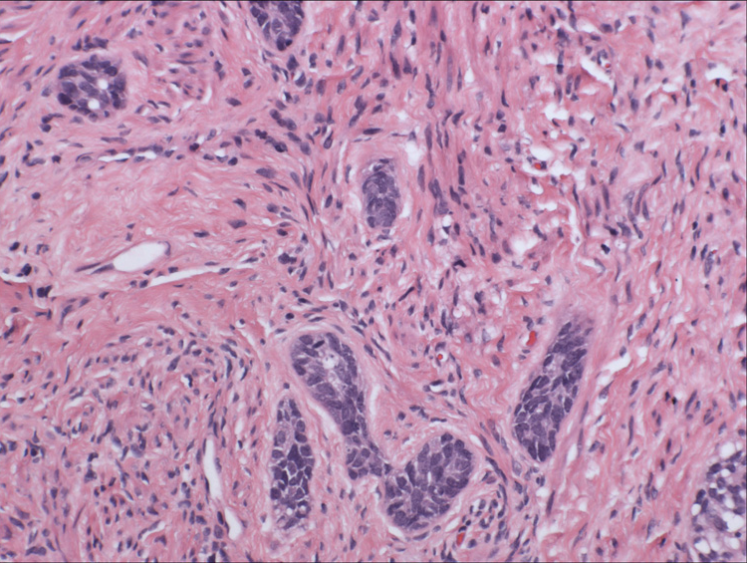
High power view of adenoid basal nests, each comprised of monomorphic, small cells with basaloid, round to oval nuclei, inconspicuous nucleoli and scant cytoplasms (hematoxylin and eosin, original magnification 60×).

**Figure 4:**
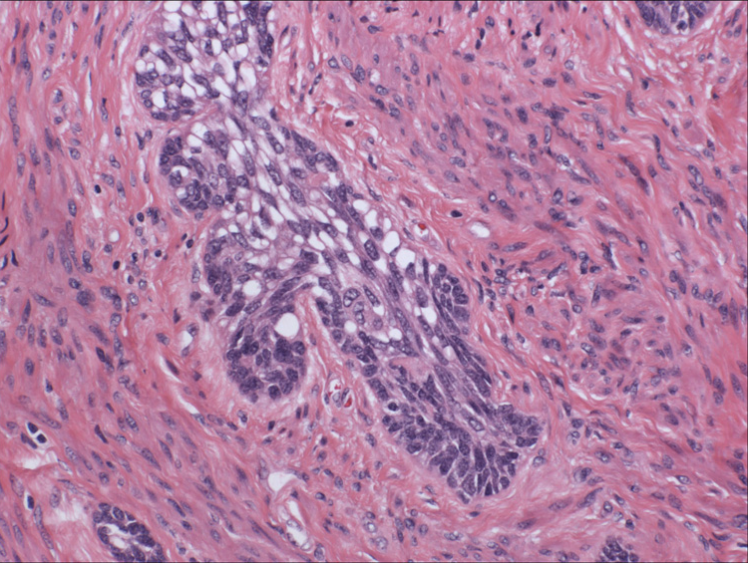
Some adenoid basal nests may show prominent clear cell change due to accumulation of cytoplasmic glycogen in constituent cells. Basaloid cells are still evident at the periphery of this nest (lower field). (hematoxylin and eosin, original magnification 60×)

**Figure 5:**
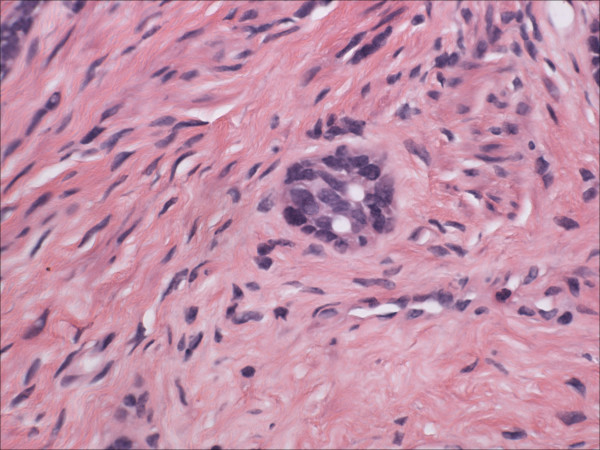
Small glandular or acinar structures such as is illustrated are not uncommonly found in adenoid basal lesions. (hematoxylin and eosin, original magnification 60×)

**Figure 6:**
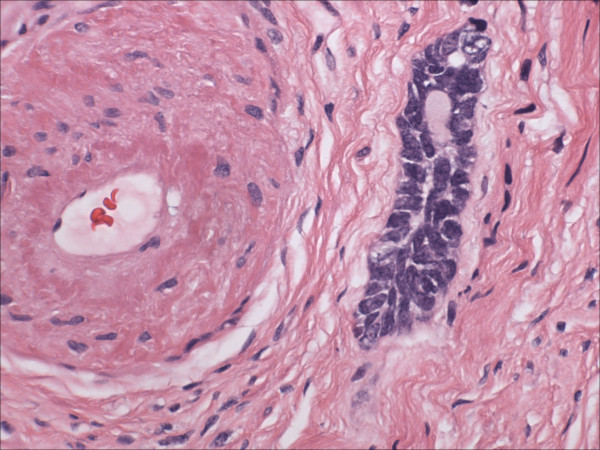
The luminal compartments of the gland-like structures may contain secretory material. (hematoxylin and eosin, original magnification 60×)

**Figure 7:**
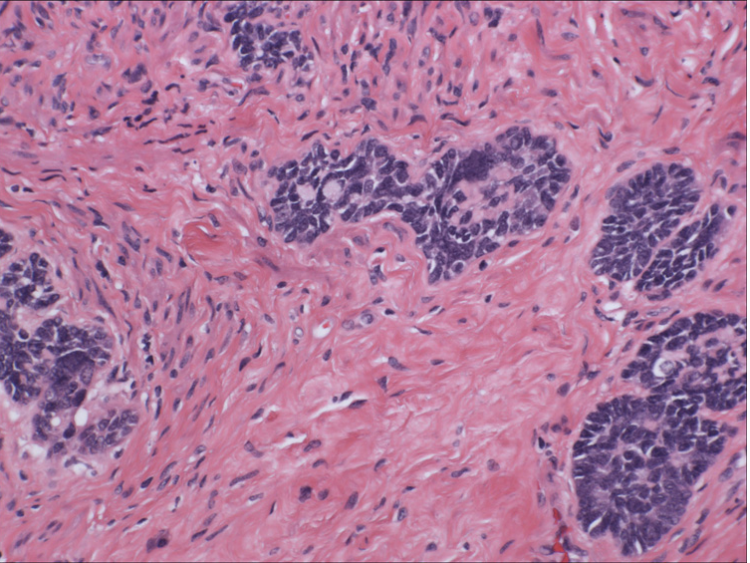
Small basement membrane-like material may be present in adenoid basal lesions. (hematoxylin and eosin, original magnification 40×)

**Figure 8:**
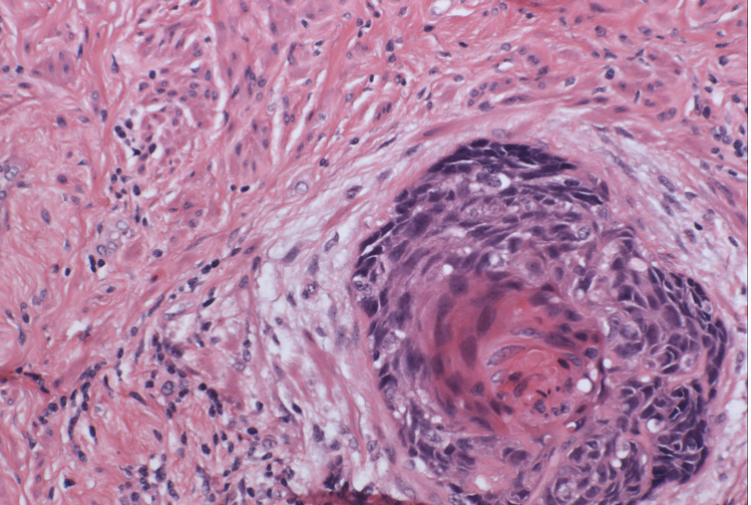
Squamous metaplasia in an adenoid basal nest. Note the basaloid cells at the periphery of the nest. (hematoxylin and eosin, original magnification 60×)

Morphologically pure ABL are not macroscopically evident. As previously noted, >90% of cases have an associated high-grade squamous intraepithelial lesion. In the subepithelial compartment is typically the incidentally discovered epithelial proliferation of ABL. The latter is comprised of multiple, small nests of basaloid epithelial cells. Scanning magnification often reveals an apparently deeply invasive process (figure 1); nests extended down into the stroma for distances that ranged from 2 mm to 10 mm (mean 4.3 mm) in one study [10] and invaded to 50–90% of the cervical stroma in another [13]. Based in part on the depth of invasion, 6 (46%) of 13 and 7 (54%) of 13 cases were International Federation of Gynecology and Obstetrics stage 1A and 1B respectively in one study [20]. Extensions into the myometrium of the isthmus have also been reported [17]. The nests are often arranged in a vague lobule-like configuration [10]. Each nest is comprised of monomorphic, small cells with basaloid, round to oval nuclei, inconspicuous nucleoli and scant cytoplasms (figure 2 and 3). Rare examples may show prominent clear cell change [9] (figure 4). The cells often palisade around the peripheral zones of the nests, in a pattern reminiscent of cutaneous basal cell carcinoma [20]. The nests are most commonly small (<100 cells) and solid, but may form glandular or even cribriform structures (figure 5). The latter were lined by cells with ciliary structures suggestive of tuboendometrioid differentiation in one case [1]. In support of their true glandular nature, another case was found to be mucicarcmine positive in these foci [14]. Secretory material may occasionally be identified within the lumen of the glandular structures (figure 6). Small basement membrane-like material may also be occasionally encountered (figure 7). Foci of squamous metaplasia may involve nests of ABL (figure 8), and such metaplasia may be so extensive as to cause the lesion to simulate an invasive squamous cell carcinoma (SCC). This potential pitfall is further magnified because the metaplastic nests are typically expanded and may contain cells showing atypia. In our experience, all cases showing this pattern have been involved by low-grade dysplasia and transitions from clefts with more clearly diagnostic foci were evident. Other authors [10] have noted that these nests typically have a faint, CAM 5.2 immunopositive rim of basaloid cells at their peripheries. These squamous nests may show prominent microcyst formation [4,13].

In their seminal study, Baggish and Woodruff emphasized that the nests immediately subjacent to the overlying epithelium seemed to be "dropping off" from it and were "well-demarcated from the adjacent stroma" [24]. Those authors have also emphasized the absence of any significant stromal reaction to the nests (figure 2), in contrast to the stromal hyalinization that may be seen in ACC. Brainard and Hart [10], however, noted that in 50% of their 12 cases, foci of "stroma edema or loosening" were present around the ABL nests and a small lymphoplasmacytic infiltrate was present around the nests in all 12 cases. Jones et al [13] also noted that "focal stromal reactions" were present in the most deeply invasive nests of their tumors, and around those nests with squamous differentiation. Layton-Henry et al [17] also illustrated in their report an adenoid basal nest that displayed squamous differentiation and an inflammatory stromal reaction. Nests of ABL should not show necrosis, perineural invasion, lymphovascular invasion or a significant desmoplastic reaction [10,20,88]; the presence of one or more of the latter features should trigger a re-evaluation of a putative diagnosis.

#### Adenoid basal lesions associated with separate malignancies

ABL may occasionally be associated with conventional malignancies within the same specimen. Morphologic transitions have been identified, albeit inconstantly, between the pure adenoid basal nests and their associated malignancies [3]. The latter may also be a focal finding within an otherwise morphologically pure adenoid basal lesion [9]. Some authors [9] have interpreted this phenomenon as examples of "divergent epithelial differentiation" in ABL. For the purpose of this review, we would refer to these as "separate" malignancies. Although this determination is undoubtedly limited by a selection bias inherent in the reported cases, ABL appears to be associated with these separate malignancies at a frequency that is much higher than would be predicted for high-grade dysplasia alone. This frequency is also much higher than would be predicted for any pseudoneoplastic proliferation of the cervix [90], which some authors have suggested ABL represents [10,85,88]. The precise frequency with which ABL is associated with separate malignancies is difficult to estimate. In their 1971 series of adenoid basal "lesions", 2 of the 5 cases reported by Baggish and Woodruff were associated with an ACC and an "epidermoid" carcinoma [23]. The recent study from Parwani et al [3] perhaps provides the best insight into this issue: these investigators searched the routine and consultation files of a large academic center (Baltimore, MD, USA) for the period between 1984 and 2004. The precise distribution of consultation versus routine cases was not outlined. Thirty-three potential cases were identified. The authors excluded from their study "typical low grade adenoid basal tumors" (n = 19), which presumably represented cases unassociated with separate cancers. Of the remaining 14 cases, 10 were retrievable and all were associated with a carcinoma of non-ABL differentiation. The associated carcinomas in 8 of the 10 cases were invasive squamous cell carcinomas (1 microinvasive), 1 was a mixture of SCC and ACC and 1 was a mixture of SCC and small cell neuroendocrine carcinoma. However, in what remains the largest series of ABL in the literature, Ferry and Scully [20], did not identify an associated carcinoma in 13 of their 14 cases (opinions differ as to whether their 14^th ^case could be considered a true ABL, vide infra). In the aforementioned study of Parwani et al [3], all 10 cases showed morphologic transitions between the ABL component and the associated conventional malignancy. The latter were distinguished from the former by virtue of their greater degree of nuclear atypia, increased mitotic activity and the presence of a stromal desmoplastic reaction. However, the invasive component, which as noted previously was most commonly SCC, also showed various degrees of "adenoid basal" differentiation ("characterized by cells in the nests with scanty cytoplasm, peripheral palisading, and focal gland formation") [3].

The relationship between ACC and ABL, separate from their aforementioned historical co-evolution, is worthy of mention. These tumors share some morphologic features, are frequently associated with cervical dysplasia, and both disproportionately affect the non-white, postmenopausal population [20]. However, their differences are more significant, and the distinction between these 2 neoplasms is of critical importance given the biologic differences between them. In contrast to patients with pure ABL, the majority of ACC patients present with postmenopausal bleeding and an apparent mass lesion [9,20,21]. Microscopically, the nests of ACC are larger, show necrosis, a higher mitotic rate and nuclear pleomorphism. ACC is also typically associated with a significant stromal reaction [9,20,21].

The aforementioned differences notwithstanding, there is at least a morphologic relationship between ACC and ABL. Varying amounts of ACC may exist in ABL and vice-versa [3,9,21,79,82,85,87,89], and morphologic admixtures and transitions have been observed between the 2 areas in tumors that are comprised of both. It has been stated that small ABL-like nests are present in up to 20% of cervical ACC [86,89]. Grayson and colleagues [9] have championed the viewpoint that ABL and ACC exist along a morphologic spectrum of basaloid neoplasms of the cervix, and that ABL may even represent a precursor to ACC (see "histogenesis and pathogenesis" below).

In addition to ACC and SCC, ABL also appears to be associated with cervical carcinosarcomas, usually admixed with an SCC, at a disproportionately high rate given its overall frequency [5,7,91]. ABL formed a major part of the epithelial component in 25% of the 8 carcinosarcomas of the cervix reported by Grayson et al [7]. Similarly, 2 of the 9 cases reported by Clement et al [91] had ABL as an epithelial component, albeit minor. These 2 studies represent the largest series of cervical carcinosarcomas in the literature.

Teramoto et al [2] recently reported 7 examples of adenosquamous cell carcinoma of the cervix showing ABL-like areas. Grayson et al [9] had previously reported similar findings, but with the adenoid basal component predominating. Small ABL-like nests have also been identified adjacent to cervical basaloid carcinomas, ie squamous cell carcinomas with basaloid features [10]

We attempted to itemize all cases in the literature in which an ABL or ABL-like lesion was identified adjacent to a separate conventional malignancy. Almost 30 cases could be retrieved [2,3,5,7,9,23,91]. The aforementioned selection bias notwithstanding, given that only 66 pure ABL have been reported, this figure appears to be substantial. However, we emphasize that the precise frequency is not readily quantifiable at present time.

#### Patient outcomes

Clinicopathologic data for 66 previously reported pure ABL are summarized in Table 1. The distribution of diagnostic and/or therapeutic measures that were administered for the 61 patients in which this data was available and/or sufficiently itemized, was as follows: conization, loop electrosurgical procedure (LEEP), or cervical excision alone (n = 11, 18%), conization or LEEP or cervical excision followed by radiotherapy (n = 2, 3.3%), conization or LEEP followed by hysterectomy ± unilateral or bilateral salpingoo-oophorectomy with or without lymph node sampling (n = 13, 21.3%), hysterectomy ± unilateral or bilateral salpingoo-oophorectomy alone with or without lymph node sampling (n = 30, 49.18%, 2 patients in this group also received chemotherapy that was unrelated to cervical pathology), hysterectomy ± unilateral or bilateral salpingoo-oophorectomy and radiotherapy with or without lymph node sampling (n = 3, 5%), Conization, bilateral salpingoo-oophorectomy, lymph node sampling and radiation (n = 1, 1.6%), dilatation and curettage (n = 1, 1.6%). Follow-up information was available for analysis in 58 (89%) of the 66 previously reported patients. 3 (5.08%) of these 59 patients died of other causes. Of the remaining 56 patients, follow-up information was itemized on each individual patient in 47 cases. None of these patients had experienced tumor recurrence, metastases or tumor-related death, with follow-up periods ranging from 1 to 156 months (mean 44.7; median 28.).

One of the cases reported by Ferry and Scully [20] has garnered a lot of attention as the only example of an alleged adenoid basal lesion that metastasized and killed the patient (within 3 months). As the authors themselves noted, the morphologic features of this tumor differed significantly from the others in their series: "the tumor cells grew slender cords that penetrated deeply into a stroma showing striking myxoid change, analogous to the morphea type of basal cell carcinoma of the skin" [20]. Hart [85] has noted that this case was "probably better classified as a high-grade invasive carcinoma with adenoid basal-like features". Ferry [88], in an editorial 9 years later, wrote that "the term ABC, without qualification, should be used only in cases with typical morphology when the tumor is composed solely of ABC (with or without associated squamous dysplasia)". We concur with the latter statement, and since we do not consider their case 14 [20] a morphologically pure ABL, it was excluded from our analysis above and elsewhere in this paper.

In summary, patients that reportedly have been diagnosed with morphologically pure ABL have had excellent outcomes, irrespective of the modality of treatment. When adenoid basal nests are associated with separate conventional malignancies, it can be anticipated that the overall prognosis will be largely dependent on the stage, grade, histologic subtype and other clinicopathologic parameters of the associated malignancy.

### Histogenesis and pathogenesis

Unfortunately, very little is known about the histogenesis or pathogenesis of ABL. In their seminal publication, Baggish and Woodruff [24] put forth 2 hypotheses to explain the histogenesis of ABL. The first was that ABL arose from the reserve cells – ie indeterminate cells with a capability for squamous or glandular differentiation – of the cervix. The second (which the others noted was "highly speculative and unlikely") was that ABL arose from misplaced ectodermal components in the cervix (i.e ABL was essentially a "variety of a basal cell or appendage lesion"). These speculations were largely based on vague morphologic similarities between their cases and adenosquamous cell carcinoma (for the first hypothesis) and cutaneous basal cell carcinoma (for the second). Over the ensuing decades, the latter hypothesis has fallen into disfavor and is generally not quoted. In contrast, there are several lines of evidence that suggest that ABL do indeed arise from a cellular population that can show multi-directional differentiation: 1) ABL have been identified in association with tumors showing glandular, squamous, biphasic epithelial (adenosquamous) and biphasic epithelial/mesenchymal (carcinosarcomas) lines of differentiation. 2) ABL tend to show squamous differentiation (see above) and glandular structures may be present. 3) Ultrastructurally, the cells of ABL are rudimentary, with sparse organelles and scattered intracytoplasmic filaments, and round to oval nuclei with indentations [6]. The composite of these features, according to some authors, causes them to bear a resemblance to normal cervical reserve cells [6]. Notably, ABL show evidence of both glandular (microvilli) and squamous (tonofilaments) differentiation even at the ultrastructural level. [6]. In addition, p63, a marker which preferentially stains the squamous (and not the glandular) component of cervical neoplasms [94], shows a marked difference in staining patterns within individual tumors, with the glandular component showing no or markedly reduced staining, and the squamous component being strongly positive [8]. An additional line of evidence that may bolster the hypothesis of the reserve cell histogenesis is the strong immunoreactivity for bcl-2 which ABL have shown to demonstrate [1,6]. The anti-apoptosis gene product, bcl-2 is normally expressed strongly in the basal layer, reserve cells and immature squamous epithelium of the uterine cervix [95]. Cviko et al [8] evaluated the proliferative index (using Ki-67 immunohistochemistry) of 5 ABL, and noted distinctive patterns of staining that suggested fundamental alterations in the cell cycle activity of these tumors. In the normal squamous epithelium of the cervix, ki-67 is expressed in the immediate parabasal layer, with no significant expression in the superficial and intermediate layers. Cviko et al [8] demonstrated that the "invasive" nests of ABL, in contrast, showed basaloid cells with variable but generally reduced expression of ki-67, in striking contrast to the squamous nests at the centers of these nests which often showed markedly increased proliferative indices. This was in striking contrast to high grade intraepithelial lesions, in which a basaloid proliferation is typically associated with a high-proliferative index. The authors concluded that ABL have a unique pattern of transitioning morphologic phenotypes associated with increasing invasion, with the overlying dysplasias showing marked proliferative indices and the deeply invasive nests showing the opposite.

Jones et al [13] investigated potential roles for the K-ras-2 oncogene and the p53 tumor suppressor gene in the etiopathogenesis ABL. None of the 5 cases they evaluated showed K-ras-2 mutations (exons 1 and 2) in polymerase chain reaction (PCR)-amplified and sequenced DNA obtained from selected sites in paraffin embedded tissue [13]. Weak immunoreactivity for the p53 protein (D0-7 clone) was identified in 4 of 5 cases whereas the expression was strong and diffuse in the 5^th^. The latter was shown to have a missense p53 point mutation (exon 7-codon 248 tryptophan). [13]. Wild-type p53 induces WAF-1 expression, thus the authors also investigated the expression of this protein as an indirect way to assess p53-related mutational changes. 2 of their 4 cases showing weak p53 expression were positive for WAF-1, whereas the 5^th ^showing strong p53 expression was negative. Senzaki et al [11] also demonstrated at the protein level, overexpression of p53 protein in an ABL. These alterations in p53 has been cited by some authors as evidence in support of the neoplastic nature of ABL [87].

Several studies have demonstrated, utilizing various modalities, a role for human papillomavirus (HPV) in the pathogenesis of ABL [1,3,5,7,8,12,13,17]. Using PCR amplified consensus primers derived from the LI genomic segment of HPV (primers designed to amplify a large spectrum of HPV types), type 16 HPV was identified in 8 of 9 cases in 2 combined studies [8,13]. In another study that utilized similar methodology [17], HPV was identified in 3 of 5 cases (1 HPV 16, 1 HPV 58 and 1 HPV MM4). Using a non-isotopic in situ hybridization (NISH) technique with digoxigenin-labeled probes for HPV types 6,11,16,18,31 and 33, 6 of 9 cases were positive (4 with HPV 16 and 2 with HPV 33), all notably in a pattern consistent with integration of the HPV genome into the host cells'[12]. For tumors associated with conventional malignancies, largely similar findings have been reported [3,5,12]. Parwani et al [3] investigated 10 such tumors using an in situ hybridization probe for HPV 16, 18 and a cocktail of different types. HPV type 16 was identified in 9 of 10 cases. PCR analysis of the 10^th ^case showed the presence of HPV type 33. Notably, both the nests of ABL and the more conventional malignancies were positive, suggesting a shared histogenetic basis. In those cases in which ABL were associated with carcinosarcomas, all 3 components (pure adenoid basal nests, sarcomatous, carcinomatous) were found to be HPV 16 positive, which further bolsters the argument of a shared histogenetic basis [5,7]. Immunohistochemical expression of P16, which is generally considered to be a surrogate marker for high-risk HPV types [96], was also detected in both the pure adenoid basal nests and the carcinomatous components in the study of Parwani et al [3]. Furthermore, as is shown in Table 1, more than 85% are associated with another HPV-mediated process, cervical intraepithelial neoplasia. Although it may be argued that the association is fortuitous, the overwhelming predominance of high-grade lesions (in contrast to low grade lesions which are more predominant in the general population) suggests a true biologic relationship, albeit one of an unclear nature. The complete immunophenotypic profile of the reported ABL is summarized in table 2.

**Table 2:** Immunophenotype of Adenoid Basal Lesions.

**Antibody**	**Number of cases tested**	**Number positive**	**Percentage positive**	**Morphologic component positive**	**Reference(s)**
EMA	14	14	100	Peri-luminary cells	9,20
CD10	1	1	100	All	1
ER**	2	1	50	All	1,11
PR**	2	1	50	All	1,11
p16	11	11	100	All	1,3
Bcl-2	2	2	100	Basaloid cells and peri-luminary cells	1,6
CEA	17	12	71	Peri-luminary cells and squamous areas	1,9,11,14,20
CAM5.2*	9	8	89	All	9
Ki-67	7	7	100	2% positive index [6]; Majority of tumor cells positive [11]; 5–50% of basaloid cells positive [8]	6,8,11
MNF116	9	9	100	All	9
CK7	13	7	54	Basaloid cells and peri-luminary cells	2,6,9,11
CK8	4	4	100	Basaloid cells and peri-luminary cells	2,6,11
CK10	1	1	100	Squamous cells	11
CK13	1	1	100	Squamous cells	11
CK14	2	2	100	Basaloid cells [11] and other "scattered cells" [6]	6,11
CK17	1	1	100	Basaloid cells	11
CK18	1	1	100	Basaloid cells and periluminary cells	11
CK19	1	1	100	Basaloid cells and peri-luminary cells	11
CK20	9	1	11	NS	9
AE1/AE3	6	6	100	All	6,20
CK902	5	5	100	All	20
α-SMA	2	0	0	NA	6,11
MSA	9	1	0	Transitional areas between a combined ABC and ACC	9
Laminin	10	1	10	Basement membrane-like material	6,9
Type IV collagen	10	1	10	Basement membrane-like material	6,9
S100	16	3	19	NS	6,9,11,20
CA125	1	0	0	NA	11
P53	6	6	100	NS	11,13
P63	6	6	100	All: reduction of staining in squamous areas and "adenoid" areas	1,8

ABC: adenoid basal carcinoma; ACC adenoid cystic carcinoma; ER: estrogen receptor; PR progesterone receptor; CEA carcinoembryonic antigen; CK cytokeratin; EMA epithelial membrane antigen; NA not applicable; SMA smooth muscle actin; MSA muscle specific actin; NS not stated.** stromal cells only were positive for ER and PR in one case [11].

*Cases reported by Brainard and Hart [10] are excluded because it is not clearly stated how many cases were tested.

In summary, ABL display a phenotype that most closely simulates immature squamous cells, squamous basal cells and/or reserve cells [p63 predominantly positive, proliferative index variable but generally low, bcl-2 positive]. This suggests an origination from (or differentiation towards) a cell that is at an early stage of a differentiation spectrum. Whether this cell is the *reserve cell*, remains to be elucidated. p53 alterations and HPV type 16 appear to play significant roles in the pathogenesis of these lesions. The finding of identical HPV types in pure adenoid basal tumors and their associated conventional malignancies, as well as the morphologic transitions between them suggests that either 1) pure adenoid basal tumors represent precursors of their associated malignancies [9] or 2) Both components are proliferating in response to the same HPV associated stimulus.

#### Nomenclature

There has been considerable controversy with respect to the precise nomenclature applied to ABL, a likely product of the continued absence of a complete understanding of their true nature and biologic potential. As noted previously, the term "adenoid cystic basal carcinoma" was initially applied by Moss and Collins [82] to describe a cervical neoplasm that would probably be considered, using contemporary criteria, a mixed ACC and ABC. The seminal 1966 report by Baggish and Woodruff [24] was captioned "*adenoid-basal carcinoma of the cervix*" and served to formally introduce the term ABC into the medical lexicon. However, the 3 lesions were described throughout the text as *"adenoid-basal lesion(s)" *and the authors even stated at the end of their text that "it would seem wise to apply the term "*adenoid-basal cell tumor*" to this unusual cervical neoplasm" [24]. The term ABC was only used in the title of their report. In a report of 5 additional cases in 1971, the aforementioned authors entitled their report "*adenoid basal lesions of the cervix*" but this time used the term ABC in their text to describe not only the new cases but the cases they previously reported [23]. These 5 cases were comprised in part of 2 cases of ABC associated with a cylindroma and an epidermoid carcinoma. The authors designated 2 of the remaining 3 cases "*adenoid basal hyperplasia*" whereas the 3^rd ^an ABC. Adenoid basal hyperplasia (ABH) was not precisely defined, and the only clinicopathologic difference between the 2 cases of ABH and the 1 case of ABC appeared to be that the basaloid proliferation invaded deep into the cervical stroma in the latter and was superficial in the former. The term ABH largely remained dormant in the medical literature until it was resurrected again in a 1985 report of van Dinh and Woodruff [21]. In that report, the authors noted that in association with "many" of their cases of ABC were areas of ABH, "consisting of buds and irregular proliferations of basal cells attached to the epithelium" and that in "no case did the basal cell buds extend more than 5 mm into the stroma" [21]. They further noted that this phenomenon was seen on 11 occasions amongst all the hysterectomies that they evaluated over a 12-year period. Three additional examples of ABH were reported by Brainard and Hart [10] in 1998. The cases of ABH were described as resembling ABC except that "the epithelial nests were smaller, and many appeared to remain attached to the adjacent squamous mucosa or endocervical clefts". In our opinion, the distinctions that have been made between ABH and ABC are so nebulous as to be nearly arbitrary. There are no solid criteria (or a solid basis for such criteria) for determining at what level of stromal invasion use of either term would be appropriate. There are no differences in clinical presentation, patient age, and certainly biologic behavior between cases of ABC and ABH [10,23]. We therefore suggest that the term ABH be discarded altogether. It serves no clinical purpose and only serves to contribute to the nomenclatural incoherence that has plagued this diagnostic entity.

Given the absence of a single case of tumor recurrence, metastasis or tumor-related death in an unequivocally pure ABL, use of the "carcinoma" designation has been questioned, most forcefully by Brainard and Hart [10,85]. These authors noted that ABL-like lesions showing atypical histologic features (i.e those associated with a separate malignancy or having otherwise atypical areas) should not be considered pure adenoid basal lesions. They proposed the term "adenoid basal epithelioma" (ABE) to describe cases previously designated ABH and histologically pure ABC [10]. The term ABE, they argued, "adequately described the composition of epithelial cells and the mixture of basaloid and glandular elements, but it avoids designation as carcinoma" [10]. They further argued that morphologically pure lesions are expected to have a clinically benign behavior, thus, applying the term ABE instead of ABC will prevent the potential over-treatment associated with a "carcinoma" diagnosis and is therefore preferable.

Grayson and Cooper [87,92], in contrast, have argued for retention of the ABC term for morphologically pure ABT. These authors argue that the aforementioned integration of high-risk HPV types [3,12], p53-related genetic changes [13], and their potential to be the dominant epithelial component of carcinosarcomas [7,91] are arguments in support of the neoplastic nature of ABL. In these authors' opinion, the term ABE "underemphasizes the lesion's potential for aggressive behavior" [87]. They suggest the term ABE be used only as a synonym for ABH. The ABC term continues to be firmly entrenched in the literature and is used in the most recent classification from the World Health Organization [93]

Our position on the nomenclature for ABL is closer to the one proposed by Brainard and Hart [10]. Although we agree with Grayson and Cooper [87,92] that the presence of high-risk HPV types in an integrated pattern as well as p53 changes argues for the *neoplastic *nature of ABL, it certainly does not argue for their *malignant *nature that would justify use of the term *carcinoma*. Although pure ABL may represent precursor lesions to other malignancies, there is no evidence to suggest that morphologically pure lesions are, in of themselves, malignant. We know of no other situation in human oncology in which the term carcinoma (without an in-situ suffix) is applied for a tumor that has not been shown to recur, metastasize or cause death. In our opinion, there is insufficient evidence at present time to expose patients with morphologically pure ABL to the ominous implications – social, psychological, medical, financial – of a "carcinoma" diagnosis. We concur with use of the designation ABE for morphologically pure ABL. As noted by Brainard and Hart [10], the term ABE acknowledges the potentially neoplastic nature of ABT, is descriptively accurate of their morphology, and most importantly, avoids use of a term that implies inherent malignant potential. However, we further recommend that the term ABE be only used if the entirety of the proliferation is evaluable, e.g in a hysterectomy specimen or in a cervical excision with negative margins. Given the aforementioned difficulties in determining the precise frequency with which ABL are associated with separate malignancies, we suggest use of the simple term "adenoid basal tumor" for lesions that extend to the margins of an excisional biopsy or which broadly involves an incisional biopsy. This may be followed by a comment explaining this description and stating that the possibility of an associated malignancy is probably low but cannot be unequivocally quantified at present. This allows the patient and her physician to make an informed decision between a hysterectomy that eliminates the risk of an unsampled associated malignancy and retention of the uterus for reproductive or other purposes. This approach, in our opinion, strikes an appropriate balance between the need to prevent over-treatment of pure lesions on one hand, and the need to ensure that the lesions are indeed pure on the other. If an ABL is associated with a separate malignancy, then all management options and primary diagnoses should be based on the stage, grade, histologic subtype and other clinicopathologic parameters of the associated malignancy. Using this approach, there is no use for the term ABC, and we agree with Brainard and Hart [10] that this designation should no longer be used.

## Summary and conclusions

Adenoid basal lesions of the uterine cervix may be associated, and occasionally show morphologic transitions with, conventional cervical malignancies, including adenoid cystic carcinoma, squamous cell carcinoma (basaloid and conventional), small cell neuroendocrine and adenosquamous carcinoma. They may also constitute the predominant epithelial component of a cervical carcinosarcoma. The determination of the precise frequency with which ABCs show this association is hampered by the inherent selection bias in the reported cases. However, this frequency appears to be substantial (>15%). Discussions about ABL should make a clear distinction between morphologically pure lesions and those associated with these separate malignancies. The biologic course of ABCs that are associated with separate malignancies is largely dependent on the clinicopathologic parameters of the associated malignancy. Morphologically pure lesions, in contrast, have a largely benign behavior, and none of the 66 reported patients have experienced tumor recurrence, metastases or tumor-associated death, irrespective of the modality of treatment. The finding of morphologic transitions between adenoid basal nests and conventional malignancies, shared HPV types in these areas, and p53 alterations, all suggest that adenoid basal lesions have some malignant potential. However, the precise magnitude of this potential is not readily quantifiable and should not dictate the management of morphologically pure lesions that are entirely evaluable. ABL continue to occupy a unique position in human oncology in which the term carcinoma (without an in-situ suffix) is applied to a tumor that has not been shown to recur, metastasize or cause death. We concur with a previous proposal that the terms *adenoid basal carcinoma *and *adenoid basal hyperplasia *should be discarded and replaced with Adenoid Basal Epithelioma (ABE). Even if all related evidence is interpreted in the most negative light, pure ABL are at worst, akin to a high grade cervical intraepithelial neoplastic lesion in that they *may *have some potential for malignant transformation and should be excised. However, based on the current state of evidence, pure ABL cannot be considered to be, in of themselves, malignant.

In our opinion, there is insufficient evidence at present time to expose patients with morphologically pure adenoid basal lesions to the ominous implications – social, psychological, medical, financial – of a "carcinoma" diagnosis. The morphologic spectrum of ABL is relatively well-defined, and any lesion that is not morphologically pure should not be designated an ABC or ABE. Furthermore, given the uncertainties regarding the frequency with which ABE are associated with separate malignancies, we wish to emphasize that the ABE designation only be applied when the tumor in question is entirely evaluable, e.g in a hysterectomy specimen or in an excisional biopsy with negative margins. Otherwise, the generic designation *adenoid basal tumor *is preferable. This approach strikes, in our opinion, an appropriate balance between the need to prevent over-treatment of pure lesions on one hand, and the need to ensure that the lesions are indeed pure on the other.

## Competing interests

The author(s) declare that they have no competing interests.

## Authors' contributions

MJR and OF co-wrote the manuscript. OF supervised the project. Both authors have read and approved the final manuscript
